# The Spatio-temporal Distribution of Japanese Encephalitis Cases in Different Age Groups in Mainland China, 2004 – 2014

**DOI:** 10.1371/journal.pntd.0004611

**Published:** 2016-04-06

**Authors:** Xiaolong Li, Shiheng Cui, Xiaoyan Gao, Huanyu Wang, Miao Song, Minghua Li, Shihong Fu, Zhi Lv, Ying He, Wenwen Lei, Bin Wang, Xiaoqing Lu, Guodong Liang

**Affiliations:** 1 State Key Laboratory of Infectious Disease Prevention and Control, National Institute for Viral Disease Control and Prevention, Chinese Center for Disease Control and Prevention, Beijing, People’s Republic of China; 2 Collaborative Innovation Center for Diagnosis and Treatment of Infectious Diseases, Hangzhou, People’s Republic of China; 3 Public Health Institute of Qingdao University, Qingdao, China; 4 Liupanshui Vocational and Technical College, Liupanshui, Guizhou, China; University of Michigan, UNITED STATES

## Abstract

**Background:**

Japanese encephalitis (JE) is very prevalent in China, but the incidence of JE among children has been greatly reduced by extensive promotion of vaccinations. The incidence of JE among adults, however, has increased in some parts of China.

**Methods/Principal Findings:**

Data on JE in mainland China, in terms of incidence, gender, and age, were collected between 2004 and 2014. We conducted spatial and temporal analyses on data from different age groups. Generally, children aged 0–15 years still represent the major population of JE cases in China, despite the gradual decrease in incidence over years. However, the incidence of JE among adults in several provinces is notably higher than the national average, especially during the epidemic waves in 2006, 2009, and 2013. The JE cases in the 0–15-year-old group are distributed mainly in the area south of the Yangtze River, with peak incidence occurring from July to September. In the adult group, especially for those over 40 years old, the JE cases are concentrated mainly in the area north of the Yangtze River. JE incidence in the adult group in September and October is significantly greater compared to the other groups. Further analysis using Local Indicators of Spatial Association (LISA) reveals that the distribution of adult JE cases in the six provinces north of the Yangtze River, between north 30–35° latitude and east 110–130° longitude, is a hotspot for adult JE cases.

**Conclusions/Significance:**

The rate of JE case increase for adults is much greater than for children and has become a public health issue. Therefore, studies on the necessity and feasibility of vaccinating adults who live in JE-endemic areas, but have never been vaccinated for JE, should become a new focus of JE prevention in the future.

## Author Summary

It is well known that children are the population most susceptible to Japanese encephalitis (JE), and the incidence of JE among children in China has been greatly reduced by extensive promotion of vaccinations aiming at children. The incidence of JE among adults, however, has increased in some parts of China. Due to a dearth of studies on JE among adults, the spatio-temporal pattern of adult JE cases is poorly understood. Here, we explore and describe the spatial and temporal distribution of JE cases observed among different age-groups in China from 2004 to 2014. The results indicate that the JE cases of 0–15-year-old group are distributed mainly in the area south of the Yangtze River; while, the adult cases, especially in the >40 age-group, are concentrated in the area north of the Yangtze River. Further cluster analysis reveals six provinces north of the Yangtze River are hotspots for adult JE cases. And the incidence of adult JE cases in these provinces is significantly higher than the national average. The increasing JE incidence among adults has become an imperative public health issue and should be attached sufficient attention.

## Introduction

Japanese encephalitis (JE) is a viral disease transmitted by mosquitoes which can lead to severe viral encephalitis. The fatality rate among JE patients is approximately 30%, while 35% of the survivors show various degrees of neurological sequelae. JE is an infectious disease of the neuron system [1–3] and is prevalent in about 24 countries and regions in Asia and Oceania. According to the latest statistics from the World Health Organization (WHO), approximately 3 billion people live in JE-endemic areas and are threatened by JE virus (JEV) infection. Nearly 67,900 JE cases occur each year, with a total incidence rate of 1.8/100,000 [2, 4].

As a mosquito-borne disease, JEV can be carried by varied mosquito species, such as Culex, Anopheles, Aedes and so on. Among these species, however, *Culex tritaeniorhynchus* is the principle vector of JEV; other mosquitoes of the genus Culex like *Culex annulirostris*, *Culex vishnui Theobald*, *Culex bitaeniorhynchus Giles*, and *Culex pipiens Linnaeus* can also act as the vector. The larvae of *Culex tritaeniorhynchus* usually live in the paddy fields, while the adults are active in the residential areas of humans. Therefore, human beings, especially those living near the rice fields becomes more easily exposed to the mosquitoes, which greatly increases the risk of JEV infection. Pigs, both domestic and wild pigs, are the major reservoirs serving as the hosts of virus amplification during the enzootic cycle of JEV transmission. So, the distance between residents and pigsties is another important factor affecting the spread of JEV in local areas, especially in some parts of Asia where pigs are kept near homes.

JE is highly prevalent in China, with several JE outbreaks occurring since 1950 [5, 6]. More than 1,400,000 JE cases were reported in China between 1963 and 1975, and the JE cases were distributed in 26 provinces, in addition to the provinces of Xinjiang, Xizang, and Qinghai [7]. The incidence of JE decreased significantly in China since the extensive introduction of JE vaccinations in 1980. The incidence rate was 3.24/100,000 in 1990 and decreased to 0.16/100,000 in 2013, which was approximately one one-hundredth the incidence rate during the period of 1960–1970 (8.32–20.92/100,000). Steps taken to control JE by vaccination have been successful [7, 8].

According to WHO data, 75% of the JE cases are children who are 0–14 years old [4]. However, a higher incidence of JE has emerged among adults in some parts of China [9]. For example, 407 JE cases were reported in 2013 in Shandong Province, along the east coast of China. Among the 407 JE cases, 109 were children 0–15 years old, while 298 cases (73%) were more than 15 years old. More specifically, 200 (49.1%) cases were more than 40 years old [10]. Therefore, the high incidence of adult JE cases has become a public health issue that should not be ignored. This study aims to elucidate the distribution of JE cases in China, from 2004 to 2014, by analyzing JE incidence data spatially and temporally.

## Materials and Methods

### Data collection and management

JE has been categorized as a notifiable infectious disease since 1950 [6]. Local JE cases are reported to the Chinese Center for Disease Control and Prevention (China CDC) by local health departments. The JE data used in this study were obtained from the China Information System for Diseases Control and Prevention (CISDCP) [11]. The study did not use patient’s medical records and all data were analyzed anonymously. These data included the reported numbers of JE cases and mortalities, and demographic information such as age and gender, from January 2004 to December 2014 in 31 provinces (cities or autonomous regions). Among the 31 provinces, only Xinjiang, Xizang, and Qinghai are free of JE.

### Analysis of epidemiological characteristics

Annual incidence and mortality, monthly incidence, and the cumulative cases from 2004 to 2014, were presented in the form of line and bar charts to illustrate the trends and season patterns of JE cases. We used a series of thematic maps, based on JE incidence data from all the China provinces, between 2004 and 2014, to analyze the spatial and temporal patterns of JE cases.

To observe differences in the JE epidemiological features of different age groups, the JE cases were grouped into three categories, namely, 0–15, 16–40, and above 40 years of age.

### Spatial cluster analysis

The Local Indicators of Spatial Association (LISA) was used to evaluate the spatial clusters of JE reported cases at the city level of six provinces north of the Yangtze River during three JE epidemic waves (2006, 2009 and 2013). By calculating the Local Moran's I coefficient, which typically ranges from –1 to 1, the spatial correlations between the data on a local area unit basis, and the average of neighboring values in the surrounding units, are revealed on LISA cluster maps [12,13].

The Z-score is used to assess the significant of observed spatial correlations, as indicated by Local Moran's I. When the Z-score is greater than 1.96 or less than –1.96, the spatial correlation of the local area units is significant (α = 0.05). A high positive Z-score indicates that the surrounding features have either similarly high values (High-High) or similarly low values (Low-Low), while a low negative Z-score indicates a significant (*P* < 0.05) spatial outlier (High-Low or Low-High) [14]. The spatial statistical analysis module of ArcGIS software (version 9.3; ESRI, Redlands, CA) was used to perform LISA analysis and identify the spatial clusters of JE cases in the JE-epidemic areas.

## Results

### Epidemiological features of JE in mainland China

#### Incidence and mortality

**Fig 1:**
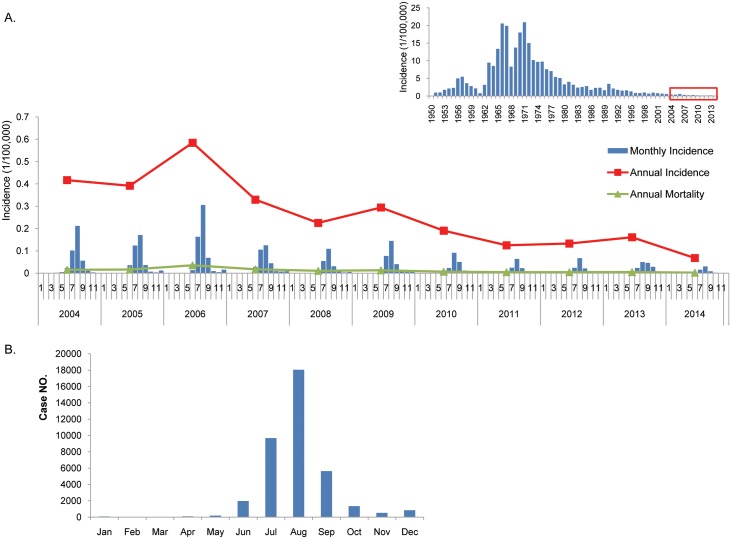
Japanese encephalitis in mainland China, 2004–2014. (A) Annual incidence and mortality, and monthly incidence of JE, from 2004 to 2014. Inserts in the right corner show the JE incidence from 1950 to 2014 [5, 8], and the research scope in this study is shown in the red box. (B) Monthly cumulative JE cases from 2004 to 2014.

From 2004 to 2014, 38,515 JE cases were reported nationwide with annual incidence rates between 0.068/100,000 and 0.5845/100,000. The average annual mortality and fatality rates were 0.0121/100,000 and 4.26%, respectively. The JE incidence and mortality rates in 2004 were 0.4171/100,000 and 0.0154/100,000, respectively, while they decreased to 0.068/100,000 and 0.0026/100,000, respectively, in 2014, suggesting a downward trend from 2004 to 2014. The JE incidence rates from 2004 to 2014 were lower than previous years (Fig 1A).

#### Gender

Among the 38,515 JE cases reported from 2004 to 2014, 23,285 are male and 15,230 are female, reflecting a male to female ratio of 1.53:1.

#### Seasonal pattern

The distribution of JE cases shows a clear seasonal pattern. JE cases peak from July to September. The highest number of cases is observed in August, whether the incidence for a given year was high (2006) or low (2014). A total of 86.7% of the JE cases occurred from July to September, while 47% occurred in August (Fig 1B).

#### Geographical distribution

**Fig 2:**
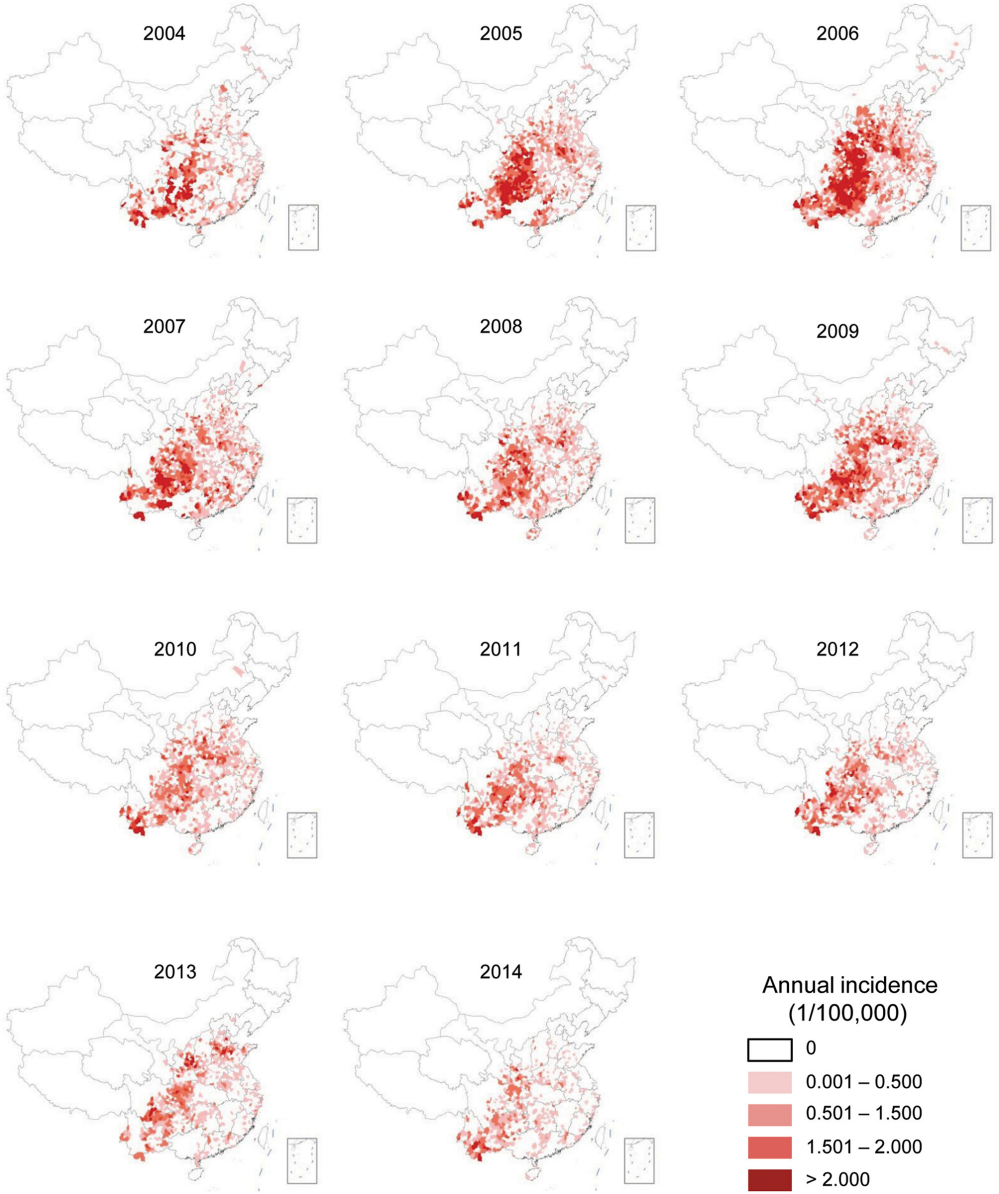
Spatial and temporal distribution of JE incidence in mainland China, 2004–2014. The JE incidence demonstrated on the map were derived from the crude rates at county level.

JE cases from 2004 to 2014 were distributed mainly in parts of South, East, and North China (Fig 2). Sichuan, Chongqing, Guizhou, and Yunnan provinces were the regions with high JE incidence. In addition, the JE incidence in several provinces in North China, such as Shanxi, Shaanxi, Henan, Shandong, Hebei, and Gansu, increased greatly during the epidemic waves of 2006, 2009, and 2013. As shown in Fig 2, the number of JE endemic areas was reduced obviously after 2008. The JE incidence rate declined nationwide, especially after 2010.

### Epidemiological features of JE cases in various age groups

#### Annual incidence and mortality rates in various age groups

To analyze incidence and mortality by age groups, JE cases from 2004 to 2014 were grouped into the three categories 0–15, 16–40, and above 40 years of age. The average annual incidence rate of JE from 2004 to 2014 was 0.27/100,000. Incidence rates for the three age groups were as follows: 1.1807/100,000 for the 0–15-year-old group, 0.0407/100,000 for 16–40-year-old group, and 0.0548/100,000 for the above 40-year-old group. The average annual mortality rate from 2004 to 2014 was 0.0121/100,000, while the mortality rates for the 0–15, 16–40, and above 40-year-old groups were 0.0485/100,000, 0.0018/100,000, and 0.0051/100,000, respectively. The number of JE cases of the three age groups, from 2004 to 2014, accounted for 84.0, 7.0, and 9.0% of the total JE cases nationwide. These results suggest that children who are 0–15 years old has the highest JE incidence in China (Table 1).

**Table 1:** Epidemiological features of JE cases in various age groups between 2004 and 2014.

Age-group	Average Annual Incidence (1/100,000)			Average Annual Mortality (1/100,000)			Peak Months	Ratio(Male:Female)
	2004–2014	2004–2008	2009–2014	2004–2014	2004–2008	2009–2014		
0–15	1.1807	1.7132	0.737	0.0485	0.0763	0.0253	Jul-Aug	1.62:1
16–40	0.0407	0.0522	0.0312	0.0018	0.0029	0.0009	Aug-Sep	1.56:1
>40	0.0548	0.0637	0.0473	0.0051	0.0069	0.0035	Aug-Sep	0.84:1

From 2004 to 2014, the JE incidence rate for the 0–15-year-old group decreased from 0.4171/100,000 to 0.068/100,000. Since the Chinese government included the JE vaccine in the Expanded Program on Immunization (EPI) in 2008, children who were 0–15 years old could get free JE vaccinations. We divided the 2004–2014 period into two periods (2004–2008 and 2009–2014), and observed the JE incidence among children 0–15 years old before and after 2008. The incidence rate is 1.7132/100,000 for the period of 2004–2008, while it falls to 0.737/100,000 for the period of 2009–2014, suggesting a obvious decrease in JE after implementing the EPI.

**Fig 3:**
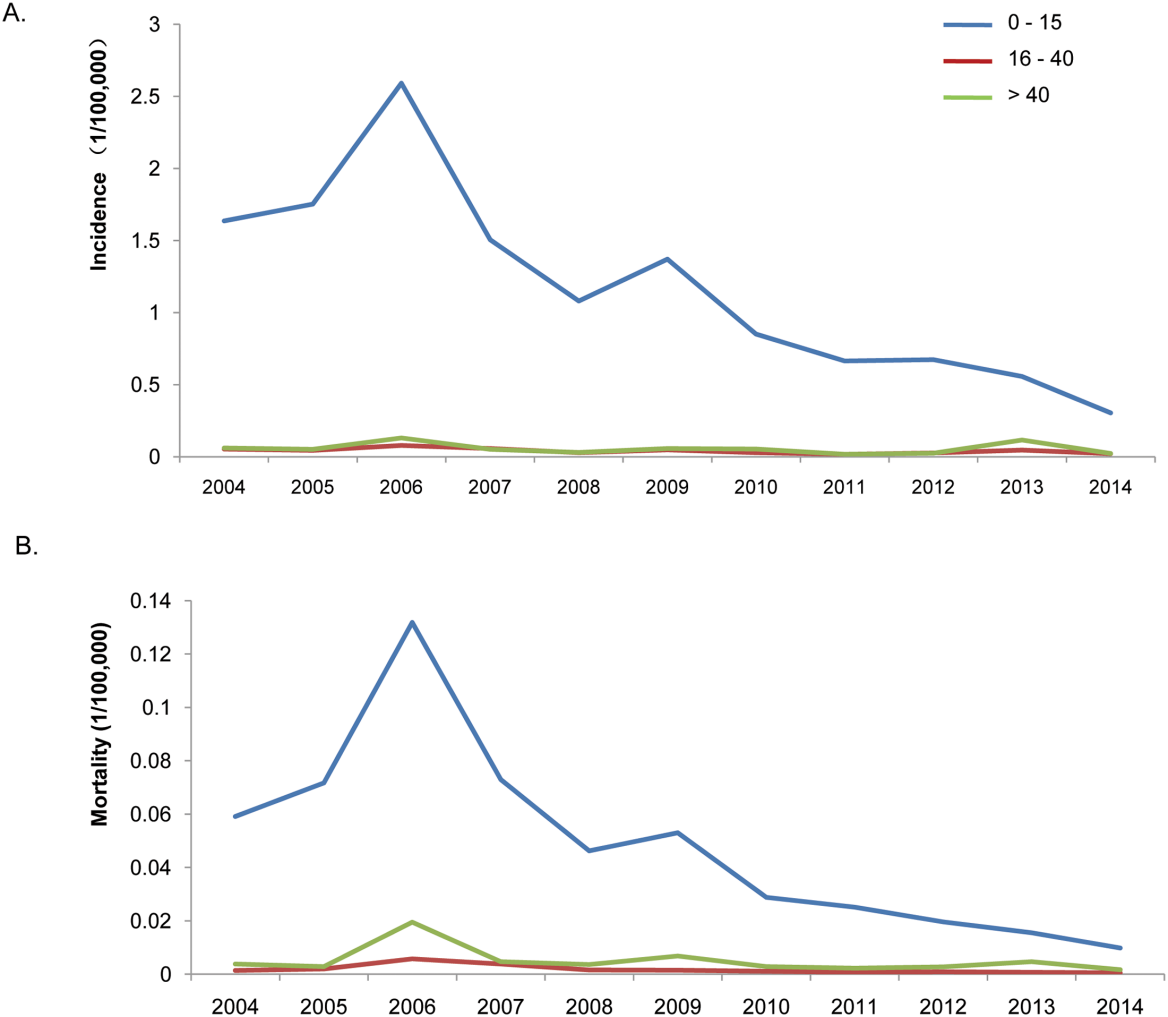
Distribution of JE cases among the age groups from 2004 to 2014. (A) JE incidences in age groups during the period of 2004–2014; (B) JE mortalities of age groups during the period of 2004–2014.

No obvious changes are observed for JE incidence in the 16–40 and the above 40 groups, between 2004 and 2014 (Fig 3A). The mortality trends of the three age groups are almost the same as the incidence trends from 2004 to 2014. Mortality of the 0–15-year-old group peaked (0.132/100,000) in 2006, and decreased thereafter. Although the mortality rates of the 16–40 and above 40-year-old groups were 0.006/100,000 and 0.019/100,000, respectively, during the epidemic wave of 2006, they were less than 0.005/100000 during the remaining years (Fig 3B).

#### Temporal distribution of JE cases in various age groups

**Fig 4:**
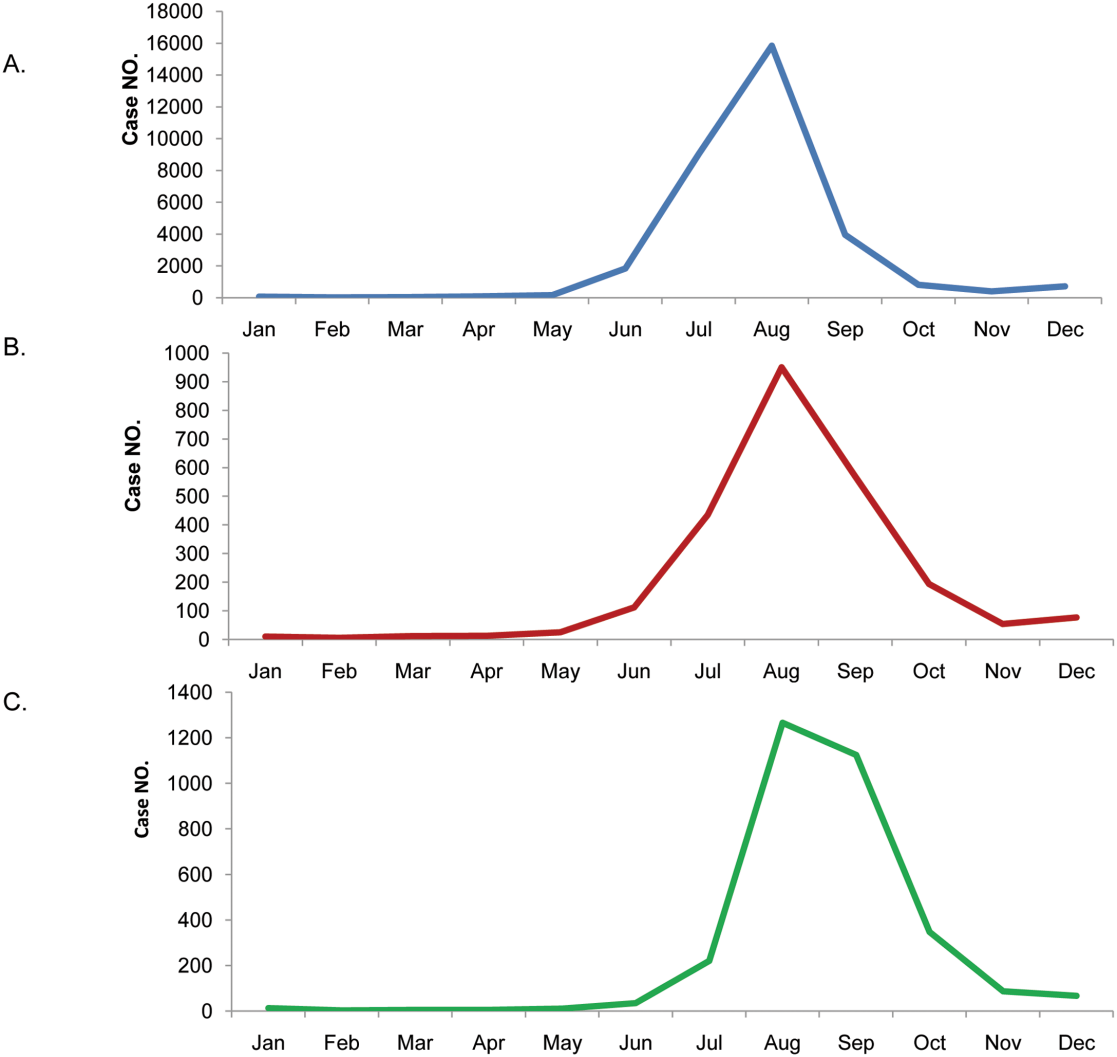
Temporal distribution of JE cases in the three age groups. (A) 0–15 years old; (B) 16–40 years old; (C) above 40 years old. Note the differences in the y-axis ranges.

The temporal distribution of JE cases among the three age groups shows that July to September are the peak months for JE, and the case numbers are highest in August. However, there are several differences in the case numbers in September and October for the three age groups (Fig 4). The number of cases for the 0–15-year-old group is highest in August, and decreases greatly in September, while the number is further reduced in October. The number of cases for the 16–40-year-old group decreases slightly in September and October. The peak number of cases of the above 40-year-old group is sustained through September, and declines in October. In September, the number of JE cases for the above 40-year-old group is greater than for the 0–15- and 16–40-year-old groups.

#### Geographical distribution of the age groups in 2004–2014

**Fig 5:**
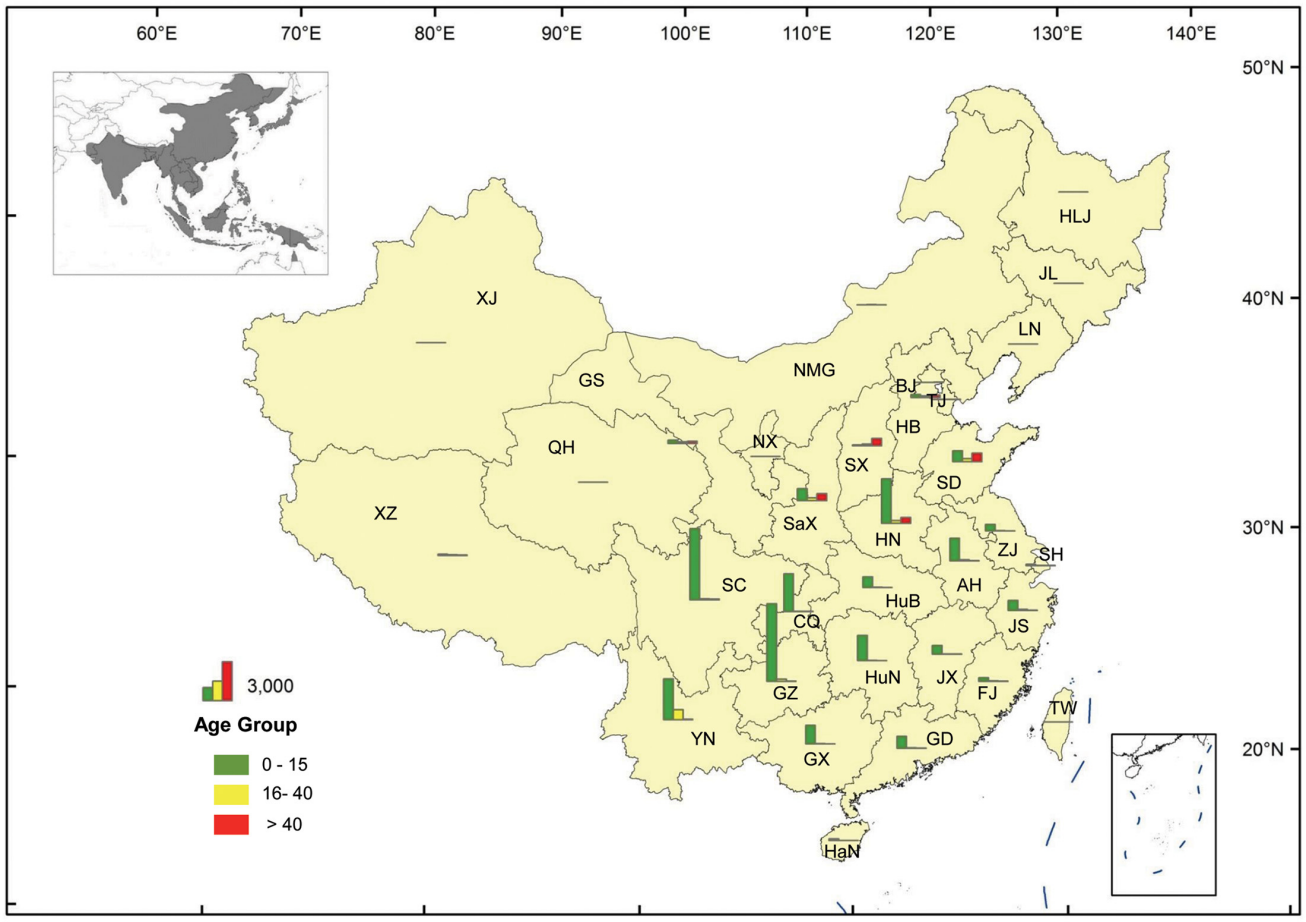
Geographical distribution of JE cases of the three age groups in 2004–2014. The grey part of the insert in the left corner indicates the prevalent region for JE cases worldwide (http://www.cdc.gov/japaneseencephalitis/maps/index.html). **Symbols:** HLJ: Heilongjiang; JL: Jilin; LN: Liaoning; NMG: Inner Mongolia; BJ: Beijing; TJ: Tianjin; HB: Hebei: HN: Henan; SD: Shandong; SX: Shanxi; SaX: Shaanxi; NX: Ningxia; GS: Gansu; QH: Qinghai; XJ: Xinjiang; XZ: Tibet; SC: Sichuan; YN: Yunnan; GZ: Guizhou; GX: Guangxi; GD: Guangdong; GX: Guangxi; HuN: Hunan; HuB: Hubei; JX: Jiangxi; JS: Jiangsu; ZJ: Zhejiang; AH: Anhui; FJ: Fujian; SH: Shanghai; TW: Taiwan.

JE cases were reported from 28 provinces in 2004–2014. Xinjiang, Xizang, and Qinghai are JE-free provinces. JE cases in the three age groups are rare in the western region next to Sichuan province (west of 100° East longitude), and the northern region next to Hebei province (north of 35° North latitude; Fig 5).

The geographical distribution of JE cases varies among the three age groups. JE cases in the 0–15-year-old group are located mainly south of the Yangtze River (south of 30° North latitude), including Yunnan, Sichuan, Chongqing, Guizhou, Hunan, and Guangxi provinces. JE cases of the 16–40 and above 40-year-old groups are distributed in the six provinces (Shanxi, Shaanxi, Shandong, Henan, Hebei, and Gansu) located north of the Yangtze River. Meanwhile, the number of JE cases in the above 40-year-old group is much greater than for the 16–40-year-old group.

#### Epidemic features of JE in three epidemic waves from 2004 to 2014

The number of JE cases in China decreased from 2004 to 2014, with three epidemic waves in 2006, 2009, and 2013 (Fig 1). The incidence rates of the 0–15-year-old group were 2.59/100,000 and 1.37/100,000 for 2006 and 2009, respectively, but decreased to 0.56/100,000 in 2013, which was even lower than the national average (1.18/100,000; Table 1) from 2004 to 2014 (Fig 3A). The JE incidence rates of the 16–40-year-old group in 2006, 2009, and 2013 were 0.08/100,000, 0.05/100,000, and 0.05/100,000, respectively, which were higher than the national average (0.04/100,000; Table 1) from 2004 to 2014. Incidence rates in the above 40-year-old group were 0.13/100,000, 0.06/100,000, and 0.11/100,000 for 2006, 2009, and 2013, respectively, which were higher than the national average of 0.05/100,000 (Fig 3).

**Fig 6:**
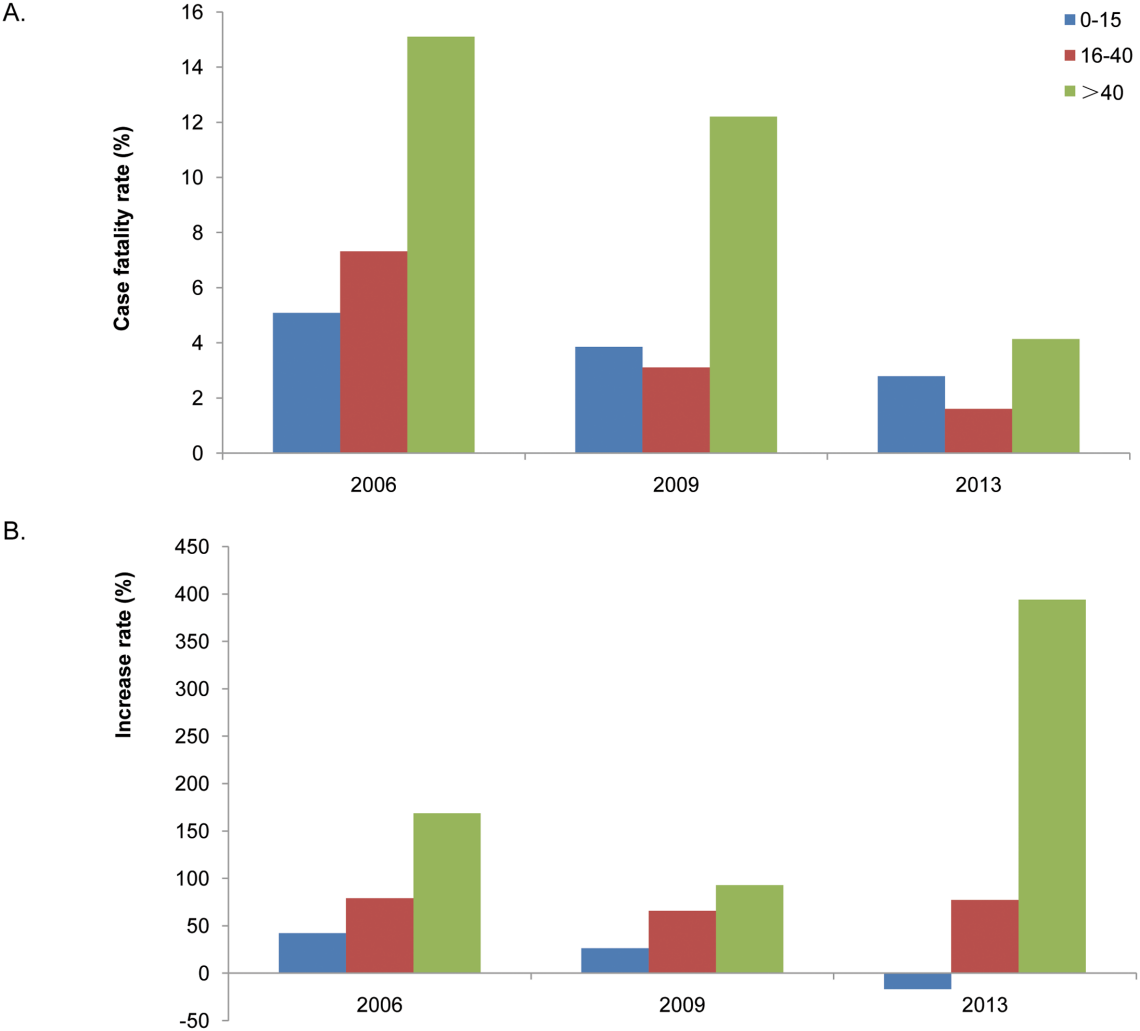
JE incidence rates for the three age groups in the three epidemic waves. (A) JE fatality rates in the three age groups; (B) Increase in JE cases compared with the previous year.

The case fatality rates of the three age groups were obviously different in 2006, 2009, and 2013. The above 40-year-old group had the highest fatality rate, which reached 15 and 12% in 2006 and 2009, respectively. Nevertheless, the JE fatality rates were relatively low in the 0–15- (2.79–5.09%) and the 16–40-year-old (1.6–7.32%, Fig 6A) groups.

We compared the numbers of JE cases of the age groups during the three years 2006, 2009, and 2013 to identify trends. The number of JE cases in the 0–15-year-old group exhibited the greatest increase (1947 cases) in 2006 when compared with 2005, and the increase rate of incident JE cases was 42.13%. The above 40-year-old group increased to 403 cases (168.62%) from 2005 to 2006. In 2009, the 0–15-year-old group experienced the highest increase in the number of incident JE cases from the previous year. The above 40-year-old group had the highest rate of increase (93%) from 2008 to 2009, which was only 26.25% for the 0–15-year-old group. The number of incident JE cases of the 0–15-year-old group decreased by 17% (255 cases) in 2013 compared to 2012, while the number increased by 394.16% (540 cases) for the above 40-year-old group (Fig 6B).

### Spatial and temporal distribution of JE cases in the above 40-year-old group

The results presented above indicate that the increase in adult JE cases, especially the adults more than 40 years old, gradually becomes the main contributor to the national increase in JE incidence from 2004 to 2014. In addition, the fatality rate of JE cases in this group is very high. These factors contribute to the urgency to investigate the temporal and spatial variation of JE incidences of the above 40-year-old group.

#### Regions with high JE case prevalence in the above 40-year-old group

**Fig 7:**
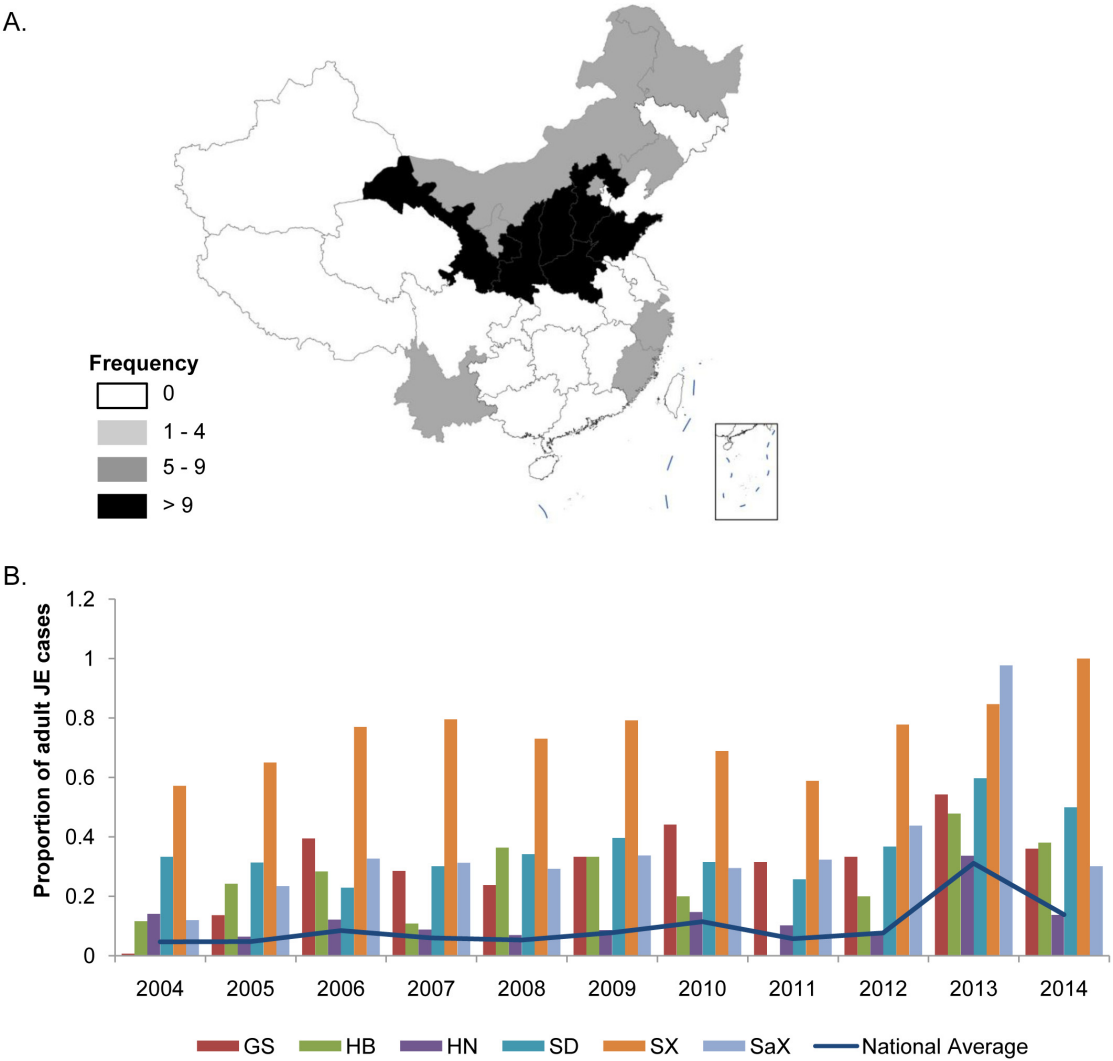
Regions with high prevalence of JE cases for the >40 group, 2004–2014. (A) Cumulative number of years when the proportion of adult cases in each province was higher than the national average, from 2004 to 2014; (B) Provinces with a proportion higher than the national average. GS, Gansu; HB, Hebei; HN, Henan; SD, Shandong; SX, Shanxi; SaX, Shaanxi.

The overall number of JE incident cases in the region south of the Yangtze River (south of 30° North latitude) is obviously greater than in the northern region (Fig 5). However, JE cases in the 0–15-year-old group are distributed mainly in the southern region, while for the group more than 15 years old, they are focused mainly in the northern region, according to the data analysis. Specifically, the cases in the above 40-year-old group are located mainly in the northern region, such as in Gansu, Shanxi, Shaanxi, Hebei, Henan, and Shandong provinces (Fig 7A).

The cumulative number of years when the proportion of adult JE cases in each province was greater than the national average is categorized into four grades (Fig 7A). Thirteen provinces have a proportion higher than the national average. Six provinces (Henan, Hebei, Shandong, Shanxi, Shaanxi, and Gansu) are higher than the national average for over nine years (the black region in Fig 7A). These regions are the areas in which cases in the above 40-year-old group are prevalent.

The proportions of JE cases for the above 40-year-old groups in the six provinces north of the Yangtze River are higher than the national average every year, from 2004 to 2014 (Fig 7B). Shanxi province has more than 50% of all the JE cases for the above 40-year-old group, which is much higher than the national average. In 2014, eight JE cases were reported in Shanxi province, and all of them were in people more than 40 years old; thus the proportion of adult cases was as high as 100%.

#### Distribution of JE cases in the above 40-year-old group during three epidemic waves

**Fig 8:**
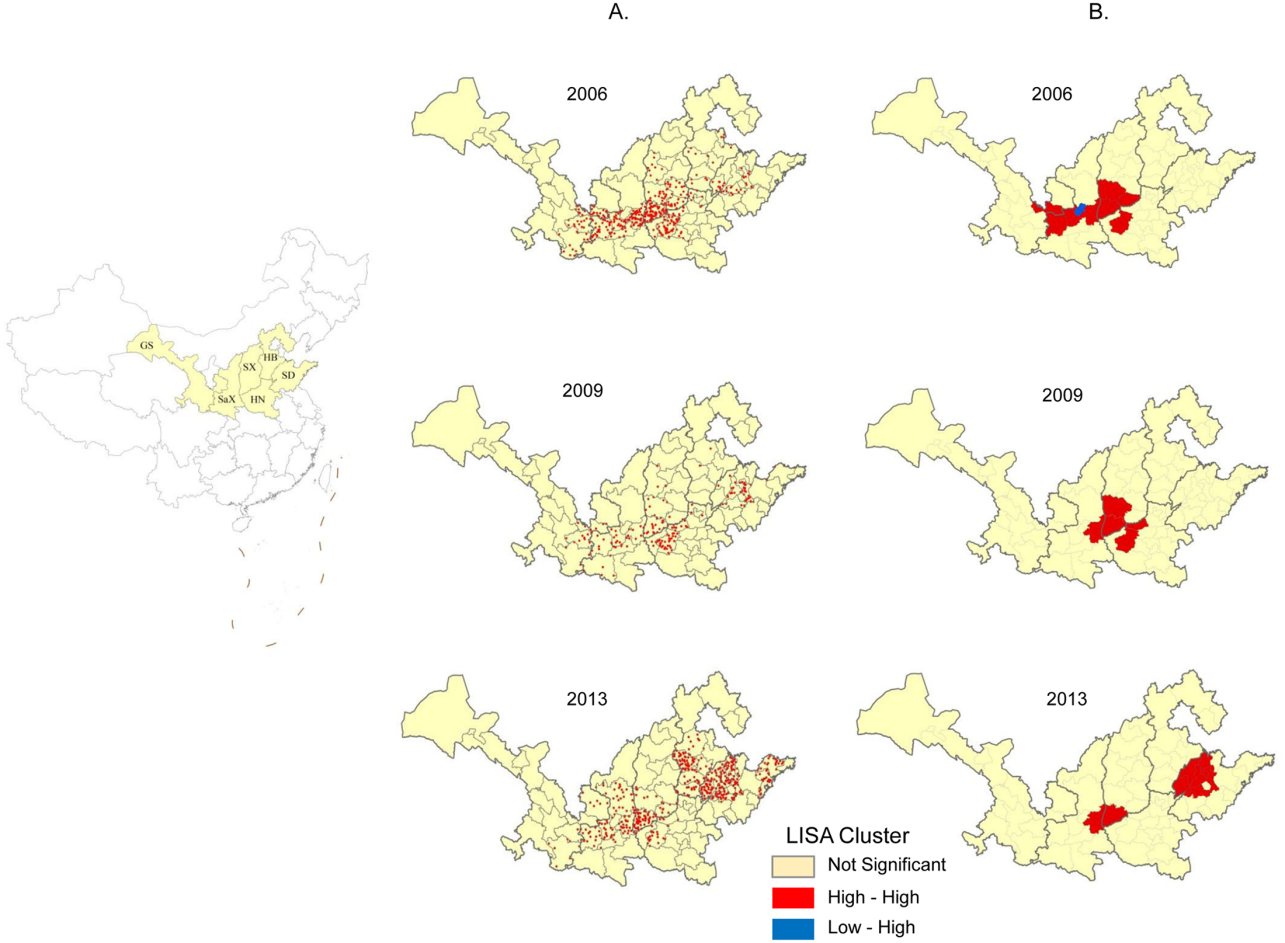
Clusters of adult JE cases in the six northern provinces during the three epidemic waves. (A) Distribution of adult JE cases; (B) LISA cluster map showing the types of clusters in color. High-High means regions with high rates are surrounded by neighbors with high rates and indicates a significant (P < 0.05) spatial cluster of high JE incidence. Low-Low represents a spatial cluster of low JE incidence values. Low-High means regions with low rates are surrounded by higher rates.

The six northern provinces were regions with a high incidence of adult JE cases, thus the case distributions in these six provinces during the three epidemic waves were analyzed. JE cases in the above 40-year-old group had a high incidence in the adjoining areas of Shanxi, Henan, and Shaanxi in 2006 (Fig 8A). JE cases were also distributed in northwestern Shandong, southern Hebei, and southeastern Gansu. And these areas presented concentrated distributions of adult cases in 2009 as well. In 2013, many of the adult JE cases were discovered in southern Hebei and northwestern Shandong, in addition to the traditionally high prevalence areas in southern Shanxi and central Shaanxi.

We analyzed clustering of adult JE cases in the six northern provinces during the three epidemic waves using Local Indicators of Spatial Association (LISA; Fig 8B). The red areas are identified as hot spots (High-High), where significant spatial clusters of high JE incidence exist. The cluster map indicates that the distribution of JE hot spots is consistent with the regions of high JE prevalence in Fig 8A, specifically focused in southern Shanxi, central Shaanxi, northwestern Henan, and northwestern Shandong.

In summary, we observe the spatial pattern for the distribution of the adult cases in the six northern provinces. JE cases are located mostly in southern Shanxi, northwestern Henan, central Shaanxi, southeastern Gansu, southern Hebei, and northwestern Shandong (110–130° East longitude, 30–35° North latitude).

#### The seasonal pattern of JE cases in the above 40-year-old group

**Fig 9:**
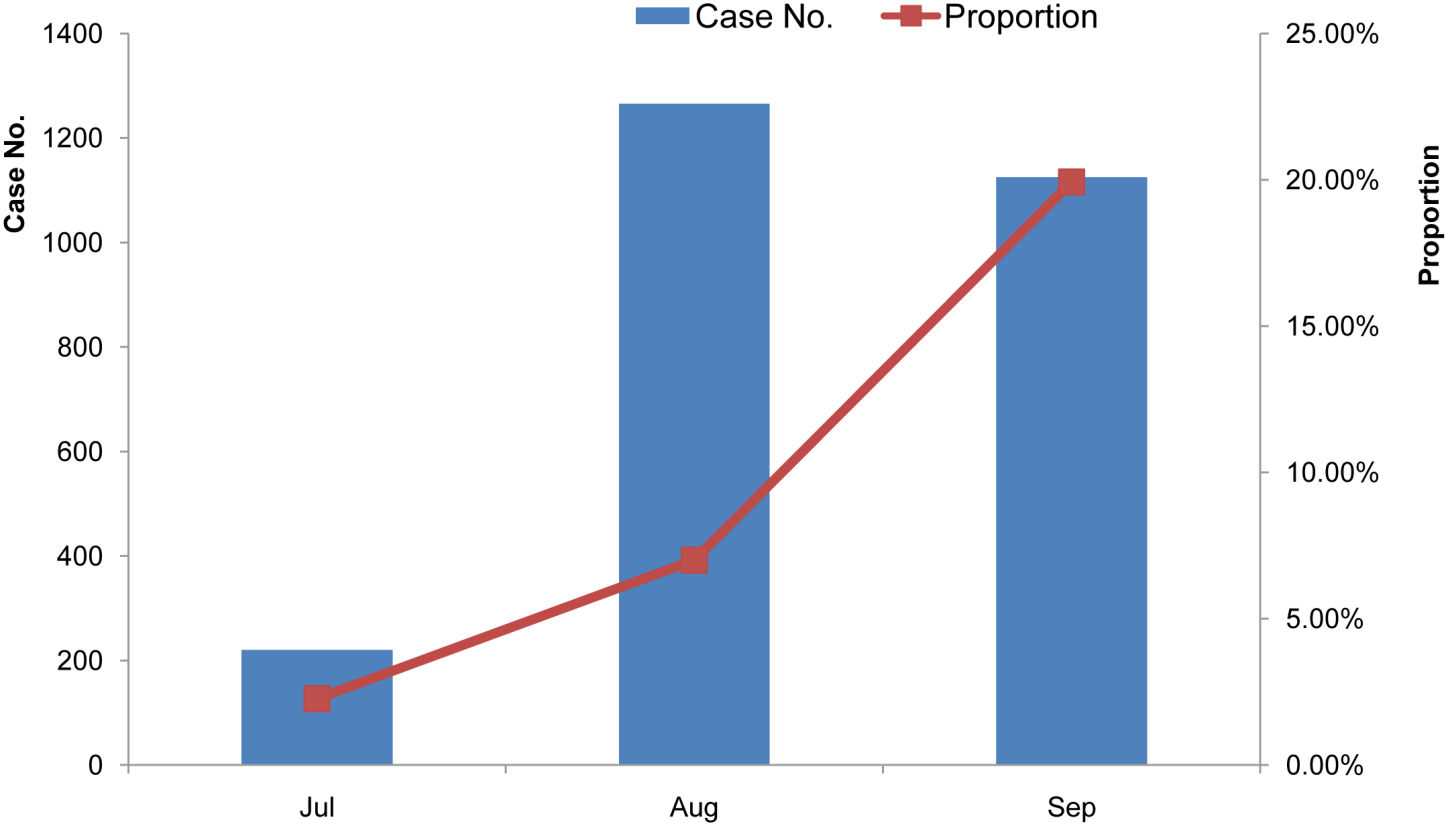
Seasonal features of JE cases in the >40 group from 2004 to 2014. Case No., the monthly cumulative JE cases in the above 40-year-old group from 2004 to 2014; Proportion, the percentage of the number of cases in the above 40-year-old group accounting for the total number of cases in a given month.

The number of cases for the above 40-year-old group peaks in August, and remains at that level in September, while it decreases sharply in October (Figs 4C and 9). However, the proportion of cases in the above 40-year-old group increases gradually in July, August, and September. The proportion of JE cases in this group in September is twice that in August.

## Discussion

The JE vaccine was included in the EPI by the China government in 2008, and, thus, children 0–15 years old could get free JE vaccinations [6,8]. Since the rate of JE vaccinations of children increased, JE incidence in children decreased significantly after 2008. For example, the JE incidence in children who were 0–15 years old in 2007 was 1.51/100,000, while it fell to 0.56/100,000 in 2013. The data suggest that the integration of the JE vaccine into the EPI significantly lowered the JE incidence in children, which was also the main reason for the recent decline of JE in China [6, 8]. There is no doubt that the incidence and mortality rates for children under 15 years old would be reduced in response to the subsequent increase of JE vaccination.

As previously mentioned, the decreased incidence of JE in China was not only attributed to the integration of JE vaccine into EPI, but the rapid development of Chinese economy in recent years also played an imperative role in reducing the JE incidence. Owe to the continually rapid economic development in past 30 years, people’s living standards have been greatly improved, which brought about a series of positive changes in hindering the spread of JEV. For example, people living in rural areas gradually moved from the simple thatched huts that cannot prevent the invasive of mosquitoes to modern buildings with screens, which notably reduced the exposure to JEV vectors than before as a result. In addition, the state invested heavily on the projects of improving water and sanitation in rural areas, and transformed the tatty latrines into clean and separate toilets in order to clear away mosquito breeding environments. Moreover, the relocation of pig farms from the residence area of the village to the places far away from the village was also conducted. These methods effectively prevented people from the exposure to mosquitoes and JEV infection and made positive contribution to reduce the JE incidence in China.

Numerous adult JE cases are found in certain provinces (Figs 5, 7 and 8). The phenomenon is identified using the nationwide infectious disease electronic reporting system, which became available in 2004. JE cases throughout the country are reported to the network of the China CDC, followed by systematic analyses [11].

Mainland China is located in the southeastern part of Asia, between north latitudes 20° (Guangdong province, the southern-most region) to 55° (Heilongjiang province, the northern-most region). Establishing 30° north latitude as a border, southern China is the region south of the Yangtze River, including some tropical regions [15]. The high average annual temperatures and heavy rainfall combined with rice-based agricultural production patterns make these areas favorable for mosquito breeding, especially for *C*. *tritaeniorhynchus*, which is the main vector for the JEV. Thus, southern China is regarded as a JE hot spot [5, 6].

Northern China, which is in the region north of the Yangtze River, (north of 30° North latitude), belongs to the north temperature zone. The four seasons are distinct in the northern part of China, with low average annual temperatures and rainfall, which are not suitable for mosquito breeding. Farmers in northern China plant drought-resistant crops such as wheat and corn, rather than rice. The density of mosquitoes is less than in southern China [6]. As a result, people living in northern China have fewer opportunities to receive the JEV passive boost immunization, compared with the people in southern China, due to climatic and geographical factors.

As mentioned above, several JE outbreaks occurred in China during the last century, particularly in the region south of the Yangtze River. In response, China successfully developed the JE-inactivated (P3 strain) and JE-attenuated live (SA14-14-2 strain) vaccines in the late 1960s and 1980s, respectively [16]. However, due to difficult economic situations, low production capacity, and limited JE vaccine distribution capability, the need for JE vaccination of children in China could not be satisfied. JE vaccine produced each year was used for emergency vaccinations of children in high JE-endemic areas [8].

Emergency vaccinations in JE-prevalent areas 30–40 years ago contributed to the relatively high JEV antibody levels in adults older than 40 (born before 1970s–1980s) in the region south of the Yangtze River. In contrast, the area north of the Yangtze River is a low JE prevalence area, with fewer outbreaks historically, and the need for fewer emergency JE vaccinations. Laboratory tests found that, in Shanxi province, which is in northern China (also one of the prevalent areas for the above 40-year-old group), the positive rate of neutralizing antibody to the JEV was 38% for the above 40-year-old group [17]. Thus, JEV antibody levels are relatively low for adults (born before the 1970s–1980s) in the region north of the Yangtze River, compared with the southern region, which is attributed to multiple climatic and historical factors. These factors contribute to the high frequency of adult JE cases in these areas.

Global warming has led to increased average annual temperatures in the region north of the Yangtze River, where the density of mosquitoes has increased accordingly. The *Culex* and *Armigeres* mosquito genera carry JEV in the northern region [6], so the opportunity for people to get infected by JEV has increased, especially for adults. Adults born during the previous century are more vulnerable to mosquitoes and JEV. They never received the JE vaccination and, thus, are easily infected by JEV. Therefore, it could be predicted that the number and proportion of JE cases in adults born during the 20th century, and not inoculated with the JE vaccine, would continue to increase. This phenomenon also occurred in other JE-epidemic areas in Asia. For example, JE vaccination of children was performed in Japan and Korea in the 1960s and 1970s, and no JE cases have been reported for children. However, adult cases in Japan and Korea continue to be reported every year, in the area north of 30° north latitude, which is similar to the spatial distribution of adult JE cases in northern China [18,19,20].

Humans are generally vulnerable to JEV. Both JE-inactivated and attenuated live vaccines are safe and effective for each age group [16, 21]. The data in this study indicate that the proportion and number of adult JE cases, as well as the proportion of incident cases among adults, are increasing annually, which would cause both human suffering and an extra burden on the government and society in general. To reduce the number of adult JE cases and the associated disease burden on society, further study is needed to determine the necessity and feasibility of vaccinating adults who live in JE-endemic areas, but have never been vaccinated with the JE vaccine. The most closely related example is JE vaccination of people traveling to JE-endemic areas. Vaccinating adults in JE-endemic areas would significantly improve the JE antibody levels and reduce the incidence of JE in adults during the 21th century.
